# Left hemothorax resulting from delayed right ventricular apical pacing lead perforation without hemopericardium, a case report

**DOI:** 10.1016/j.ijscr.2022.106924

**Published:** 2022-03-09

**Authors:** Natasha Topoluk, Hannah Kieffer, Heather Sutter, Evgueni Fayn, Paul S. Pagel, G. Hossein Almassi

**Affiliations:** a Department of Anesthesiology, Medical College of Wisconsin, Milwaukee, WI, United States of America; b Medical College of Wisconsin, Milwaukee, WI, United States of America; c Department of Surgery, Division of Cardiothoracic Surgery, Medical College of Wisconsin, Milwaukee, WI, United States of America; d Division of Cardiology, Clement J. Zablocki Veterans Affairs Medical Center, Milwaukee, WI, United States of America; e The Anesthesia (PSP) Service, Clement J. Zablocki Veterans Affairs Medical Center, Milwaukee, WI, United States of America; f Section of Cardiothoracic Surgery, Clement J. Zablocki Veterans Affairs Medical Center, Milwaukee, WI, United States of America

**Keywords:** Pacemaker lead, Hemothorax, Hemopericardium, Cardiac perforation

## Abstract

**Introduction and importance:**

Right ventricular pacemaker lead perforation is a rare but well documented complication of pacemaker implantation. Lead perforation can cause an array of symptoms ranging from none to hemodynamic instability and tamponade. In previously reported cases, lead perforation has always been able to be confirmed by imaging, with computed tomography (CT) scan considered to be the gold standard diagnostic imaging modality.

**Case presentation:**

An 80-year-old male underwent uncomplicated implantation of a dual chamber pacemaker for sick sinus syndrome as an outpatient. Thirty-nine days later, the patient presented to the emergency department complaining of new-onset, left-sided, pleuritic chest pain. He was found to have unilateral hemothorax and abnormal pacemaker lead interrogation. Pacemaker lead perforation was suspected but not confirmed with imaging. Lead perforation was only identified after surgical exploration.

**Clinical discussion:**

This patient had multiple risk factors for pacemaker lead perforation. However, imaging, including CT scan was unable to confirm perforation. The presence of an otherwise unexplained left hemothorax strongly suggested that surgical intervention was indicated. The lead perforation was subsequently confirmed with subxiphoid exploration of the pericardial space. The mechanism of lead perforation resulting in hemothorax in this case is not straight forward, as no direct communication between the pericardial and pleural spaces was identified. However, previously described visceral pericardial self-sealing may contribute to the small pericardial accumulation described herein.

**Conclusion:**

This patient's presentation and clinical course underscore the importance of maintaining a high index of suspicion for pacemaker lead perforation despite a lack of confirmation with imaging.

## Highlights

• Cardiac perforation by pacing lead results in hemopericardium.
• Isolated hemothorax from lead perforation without hemopericardium is highly unusual.
• Computed tomography is the most sensitive diagnostic imaging test.
• CT imaging can be inconclusive in delayed lead perforation.
• Surgical exploration is required in select cases of delayed RV perforation.

## 1 Introduction and importance

Lead perforation is an unusual complication of pacemaker implantation [1], [2] that occurs more frequently in right ventricle (RV) than the right atrium [1], [3]. Lead perforation is temporally defined as acute (less than 24 h after implantation), sub-acute (more than 24 h but less than 30 days), or delayed (greater than 30 days). The in-hospital mortality associated with lead perforation is 1% [1]. Acute perforation carries the greatest mortality and most often presents with pericardial effusion or tamponade. The sub-acute and delayed forms of lead perforation account for less than one-quarter of cases and are highly variable in presentation, ranging from completely asymptomatic to frank tamponade [1], [4], [5]. Lead perforation rarely causes left hemothorax regardless of its acuity [3]. Abnormal findings during pacemaker interrogation raises the possibility of perforation [5], [6]. Computed tomography (CT) has been established as the most sensitive imaging technique for the diagnosis of lead perforation [3], [7]. We report a case of RV apical lead perforation presenting as left hemothorax. In our case, CT did not reveal the lead perforation and surgical exploration of the pericardial space was required to identify it. This work has been reported in line with the 2020 SCARE criteria [8].

## 2 Case presentation

**Fig. 1:**
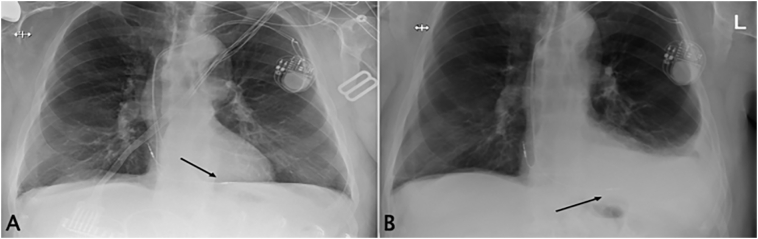
Chest radiograph confirming RV lead placement immediately after pacemaker placement (left panel; A) and when the patient returned to the hospital with pleuritic left chest pain (right panel; B); no lead migration was noted, but a large pleural effusion was present.

**Fig. 2:**
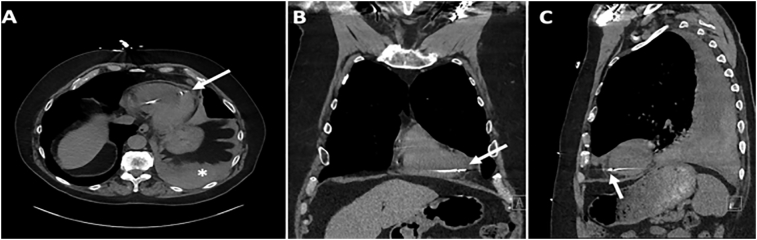
Chest computed tomography in the axial (left panel; A), sagittal (middle panel; B), and coronal (right panel; C) planes showing RV pacing lead, intact pericardium, and no effusion; the asterisk (*) denotes the pleural effusion.

An 80-year-old, 112 kg, 188 cm man with chronic atrial fibrillation (treated with apixaban), urothelial carcinoma, and metastatic prostate cancer with pulmonary nodules underwent implantation of a dual chamber pacemaker for sick sinus syndrome as an outpatient at the authors' institution (Fig. 1A). The procedure was uncomplicated. Thirty-nine days after the pacemaker was placed, the patient presented to the emergency department complaining of new-onset left-sided pleuritic chest pain. Pertinent history was unchanged from previously described; family, social and other medication histories were unremarkable. A bedside ultrasound examination indicated that the patient had a left pleural effusion. A chest radiograph confirmed this finding (Fig. 1B). The patient was admitted for further diagnostic evaluation and treatment. Pacemaker interrogation demonstrated transient increases in impedance and pacing threshold with dropped R waves. Right ventricular lead perforation was suspected as a cause for left pleural effusion. The patient underwent non-contrast CT imaging, which verified the presence of a left pleural effusion (Fig. 2A, B, and C), but lead displacement, pericardial effusion, and perforation of the RV or the pericardium were not apparent. Despite these negative findings, cardiothoracic surgery was consulted for a possible lead perforation. Transthoracic echocardiography showed normal RV and left ventricular dimensions and function. There was no evidence of a pericardial effusion. Color Doppler blood flow mapping did not show unusual flow from the RV into or within the pericardium. The patient underwent a thoracentesis 48 -h after apixaban was discontinued. A total of 1.2 L of blood was drained from the left hemithorax, after which the patient's symptoms improved. Analysis of the pleural fluid was consistent with a non- malignant, bloody effusion; Gram stain and cultures were negative.

The patient was taken to the operating room by the senior author (GHA) for a subxiphoid pericardial window and lead removal for presumed RV perforation and placement of a new pacing RV lead. During surgery, the pericardium appeared to be intact without a puncture or a protruding pacemaker lead. The pericardium was incised, revealing a minimal amount of bloody effusion. No palpable lead or other abnormality was noted along the surface of the heart including the apex during gross inspection. The heart was displaced to the right using a laparotomy sponge to expose the apex more clearly, and a lead was seen to be protruding from the RV apex without evidence of active bleeding or thrombus. The protruding lead was cut, and the RV repaired. No direct communication with the left pleural space could be identified. The incision was closed. The electrophysiologist (EF) then proceeded to extract the remainder of the RV pacing lead and insert a new pacemaker lead. The patient tolerated the procedure well. Serial postoperative chest radiographs were obtained to monitor for pleural effusion recurrence, which did not occur. The remainder of the patient's hospital course was unremarkable, and he was discharged on the fourth postoperative day. At the latest follow-up telephone visit (January 2022), patient remains active and doing well and expressed his appreciation for the care he received seven months earlier.

## 3 Clinical discussion

Our patient's complaint of new-onset pleuritic chest pain resulting from a hemothorax is an uncommon presentation for delayed lead perforation [9], which is more often characterized by signs and symptoms associated with pericardial fluid accumulation (e.g., dyspnea, atypical chest pain) or diaphragmatic irritation (e.g., persistent hiccups) [9]. The most consistent sign suggesting the possibility of lead perforation is abnormal pacemaker function during interrogation [5], [6] including loss of capture or alterations in impedance and sensing [3], but imaging is usually required to confirm the diagnosis [3], [6], [9]. As observed in our patient, a chest radiograph does not always reliably demonstrate lead migration or perforation. Similarly, transthoracic or transesophageal echocardiography has limited utility in this setting because a pericardial effusion from a lead perforation is not always present [5], [6]. In contrast, CT is generally regarded as the imaging technique of choice for confirming lead perforation [5], [6], [7], and three previous reports of RV lead perforation causing left hemothorax were confirmed using this modality [1], [3], [4]. Additional reports of cardiac foreign bodies have been confirmed with CT imaging, and have been directly visualized during surgical exploration due to pericardial and/or pleural penetration [10]. However, CT scans obtained in our patient were unable to clearly identify his RV apical perforation, and its presence was not immediately apparent, though ultimately confirmed, during surgery.

Our patient had two major risk factors for lead perforation: 1) placement of an active fixation lead in the RV apex and 2) resumption of systemic anticoagulation (apixaban) three days after the lead was placed, but other risk factors (e.g., female sex, age greater than 80 years, use of corticosteroids) were absent [1], [2], [9]. Nevertheless, the possibility of lead perforation was not considered as a cause of the left pleural effusion until interrogation of the pacemaker was performed and the results were abnormal. Our patient's history of two primary cancers and pulmonary nodules initially raised the concern that his left pleural effusion was malignant or infectious in origin, but the thoracentesis findings excluded these etiologies. The imaging studies were nondiagnostic and also lowered our suspicion for lead perforation, as we could not explain the mechanism responsible for the hemothorax with an intact RV and pericardium. A large unilateral exudative effusion resulting from pacemaker-associated post-cardiac injury syndrome was reported several weeks after pacemaker implantation [11], but lead perforation and hemothorax were absent in this case, and this possibility was excluded in our patient as a result. After thoracentesis confirmed the presence of hemothorax, suspicion for lead perforation was deemed to be sufficiently great enough to warrant surgical exploration in accordance with the American Heart Association and Heart Rhythm Society guidelines [12], [13]. Delayed lead perforation rarely causes hemodynamic instability, but this form of perforation is associated with increased risk of mortality, which is why surgical assistance in complicated cases is recommended. Surgical consultation usually occurs when CT or other imaging establishes the diagnosis of lead perforation, but the CT scans (Fig. 2) in our patient were unable to definitively identify the perforation. Nevertheless, the presence of otherwise unexplained left hemothorax in our patient with risk factors for delayed lead perforation strongly suggested that surgical intervention was indicated.

We used a subxiphoid surgical incision for this patient. This approach provides easy access to the pericardium and the right ventricle for excision of the protruding lead and repair of the RV apex. In patients with ongoing bleeding and hemodynamic compromise, a median sternotomy incision is the preferred option for stabilization of the patient and control of bleeding site.

The mechanism responsible for delayed lead perforation has been proposed as a gradual process in which the combination of contracting myocardium, reactive fibrosis, and the lead itself are thought to “self-seal” the RV and the investing visceral pericardium, thereby limiting rapid fluid accumulation and the size of the resulting effusion [5], [6], [9]. A small volume of bloody pericardial fluid was encountered during surgery consistent with this mechanism, but it is unclear how such a process could account for the large hemothorax observed in our patient, as no injury to the parietal pericardium was noted during surgery. It is highly unlikely that our patient had an anatomic variant connecting the pericardial and pleural spaces, as such an anomaly is exceptionally rare, should have been easily detected on thoracic imaging and direct inspection, and should be associated clinically with recurrent pleural effusion. Studies in rodents, rabbits, and dogs demonstrated that natural pores exist between the pericardial and pleural space [14], [15], which may allow movement of fluid from the pericardium to the pleural during acute fluid accumulation events, but the presence of such pores or whether they might be capable of unidirectional transfer of such a large fluid volume has yet to be confirmed in humans.

## 4 Conclusion

In summary, our patient's presentation and clinical course underscore the importance of maintaining a high index of suspicion for pacemaker lead perforation despite a lack of confirmation with imaging.

## Provenance and peer review

Not commissioned, externally peer-reviewed.

## Source of funding

This research did not receive any specific grant from funding agencies in the public, commercial, or not-for-profit sectors.

## Ethical approval

NA.

## Consent

Written informed consent was obtained from the patient for publication of this case report and accompanying images. A copy of the written consent is available for review by the Editor-in-Chief of this journal on request.

## Research registration (UIN: for case reports detailing a new surgical technique or new equipment/technology)

NA.

## Guarantor

G. Hossein Almassi, MD (Senior author)

## CRediT authorship contribution statement

1. Natasha Topoluk: Conception and design, acquisition of data, Drafting and revising the work, agreement to be accountable for all aspects of the work, Approval of the final version for submission and publication.

2. Hannah Kieffer: Conception and design, acquisition of data, Approval of the final version for submission and publication.

3. Heather Sutter: Conception and design, acquisition of data, Approval of the final version for submission and publication.

4. Evgueni Fayn: Conception and design, acquisition of data, Approval of the final version for submission and publication.

5. Paul S Pagel: Conception and design, acquisition of data, Review and critical revision of the work, agreement to be accountable for all aspects of the work, Approval of the final version for submission and publication.

6. G. Hossein Almassi: Corresponding Author, Conception and design, acquisition of data, Review and critical revision of the work, agreement to be accountable for all aspects of the work, Approval of the final version for submission and publication.

## Declaration of competing interest

Authors have no conflict of interest related to this manuscript.
